# The radiation of cynodonts and the ground plan of mammalian morphological diversity

**DOI:** 10.1098/rspb.2013.1865

**Published:** 2013-10-22

**Authors:** Marcello Ruta, Jennifer Botha-Brink, Stephen A. Mitchell, Michael J. Benton

**Affiliations:** 1 School of Life Sciences, University of Lincoln, Lincoln LN2 2LG, UK; 2 Karoo Palaeontology, National Museum, PO Box 266, Bloemfontein 9300, South Africa; 3 Department of Zoology and Entomology, University of the Free State, Bloemfontein 9300, South Africa; 4 School of Earth Sciences, University of Bristol, Bristol BS8 1RJ, UK

**Keywords:** cynodonts, disparity, diversity, morphospace, phylogeny, rates

## Abstract

Cynodont therapsids diversified extensively after the Permo-Triassic mass extinction event, and gave rise to mammals in the Jurassic. We use an enlarged and revised dataset of discrete skeletal characters to build a new phylogeny for all main cynodont clades from the Late Permian to the Early Jurassic, and we analyse models of morphological diversification in the group. Basal taxa and epicynodonts are paraphyletic relative to eucynodonts, and the latter are divided into cynognathians and probainognathians, with tritylodonts and mammals forming sister groups. Disparity analyses reveal a heterogeneous distribution of cynodonts in a morphospace derived from cladistic characters. Pairwise morphological distances are weakly correlated with phylogenetic distances. Comparisons of disparity by groups and through time are non-significant, especially after the data are rarefied. A disparity peak occurs in the Early/Middle Triassic, after which period the mean disparity fluctuates little. Cynognathians were characterized by high evolutionary rates and high diversity early in their history, whereas probainognathian rates were low. Community structure may have been instrumental in imposing different rates on the two clades.

## 1. Introduction

The origin and rapid initial radiation of species-rich clades may be linked to key evolutionary innovations, opportunistic expansions during periods of diminished competition and/or niche exploration after large-scale biological crises [1]. The time following these crises may witness patterns of diversification that reset evolutionary clocks and/or re-build ecosystems [2,3]. The most catastrophic crisis on record—the Permo-Triassic mass extinction event (PTME), some 252 million years (Myr) ago—challenged the surviving organisms with extensive global warming, acid rain and forest loss [4–6]. The response of terrestrial vertebrates to the PTME is the focus of novel enquiry [7–9]. Some groups (temnospondyl amphibians; therocephalian synapsids; procolophonid parareptiles [10–12]) passed through the PTME at low diversity and expanded in the Triassic. Others (anomodont therapsids [13]) were diverse and abundant in the Late Permian, went through a bottleneck at the PTME and recovered in the Triassic.

**Figure 1.**
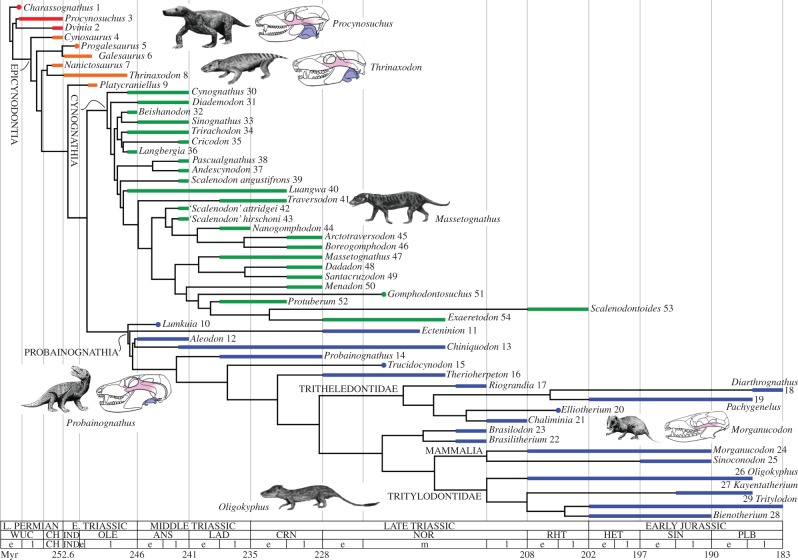
Cynodont tree plotted onto a stratigraphic scale; rectangular bars or dots show the known observed ranges of taxa; e, early; m, middle; l, late; each taxon is identified by a number, for ease of comparisons with the plots in figure 2*a–c*. For stage abbreviations, see §2d.

Cynodont therapsids exemplify a group of land vertebrates that survived the PTME and diversified extensively in the Triassic. In addition, they offer an excellent model for studying clade diversification leading up to the origin of a successful and iconic vertebrate radiation—the mammals. Cynodonts gave rise to the mammals in the Jurassic. Their skeletal anatomy documents in exquisite detail major skeletal changes in the braincase, lower jaw, teeth and limbs that foreshadow the mammalian ground plan [14–19]. Among the earliest recorded genera are *Charassognathus* and *Procynosuchus*, both from the Wuchiapingian *Tropidostoma* Assemblage Zone of the Karoo Basin in South Africa [20–22]. By the End-Permian, basal cynodonts had a wide distribution. As an example, *Procynosuchus* has been found in South Africa, Tanzania, Zambia, Russia and Germany [15,19,22,23]. A number of the more derived early cynodonts (epicynodonts; figure 1) showed traits regarded as mammalian hallmarks, such as those associated with food collecting/processing, including differentiated teeth and an enlarged dentary [14–19]. The Early Triassic *Thrinaxodon* featured a mammal-like secondary palate and an expanded zygomatic arch [16]. The epicynodonts first appeared in the Late Permian. Some lineages survived into the Triassic, but no known genera crossed the Permo-Triassic boundary (PTB) [8,21–24]. Increasingly, active lifestyles in the Triassic allowed cynodonts to begin to exploit diverse food supplies. Novel structural and functional changes ensued, including a differentiated dentition and a more efficient oxygen-pumping mechanism (inferred from their secondary palate and presumed presence of a diaphragm) [14–19,25]. During the cynodont–mammal transition, the lower jaw underwent remarkable modifications: its posterior bones migrated backward and upward into the middle ear, thus supplementing the reptilian stapes with the mammalian malleus and incus [17–19]. At the same time, a shift took place in the jaw joint pattern (from quadrate–articular to squamosal–dentary). A sprawling-to-erect postural change also occurred, such that the femur rotated from a lateral to a parasagittal position (similar modifications occurred in parallel in other synapsid clades and in archosaurs [19,26,27]). Although Mesozoic mammals probably still laid eggs, most derived characters of mammals had already been established in the Triassic [14–16,19,28,29]. Derived cynodonts, or eucynodonts, became major components of land vertebrate faunas worldwide immediately after their first appearance by the end of the Early Triassic. They included two groups: the largely herbivorous cynognathians and the mainly carnivorous probainognathians (figure 1). Several cynodont lineages died out in the Late Triassic, but the herbivorous tritylodonts and the omnivorous as well as insectivorous tritheledonts survived into the rest of the Mesozoic [30,31] (for geographical, stratigraphic and geological data, see electronic supplementary material, datasets S1 and S11). Here, we explore the nature of the cynodont radiation with analyses of disparity and evolutionary rates, and discuss it in the context of diversification models near the ancestral roots of mammals [32,33].

## 2. Material and methods

### (a) Phylogenetic analyses

We built a data matrix (electronic supplementary material, dataset S2) of 150 discrete skeletal characters (see electronic supplementary material, dataset S3) coded for 52 cynodonts (representing over 68% of recorded Permian–Lower Jurassic cynodont diversity, excluding dromatheriids and all post-trytilodont taxa) and the two early mammals *Morganucodon* and *Sinoconodon*. The matrix includes three early-diverging taxa, six basal Epicynodontia, 18 Probainognathia and 25 Cynognathia. The excluded taxa are based mainly on incomplete material (see electronic supplementary material, datasets S9 and S10), and so could not be coded meaningfully.

We carried out three maximum-parsimony analyses: the first with all characters equally weighted and unordered, the second with characters reweighted according to the best fit (i.e. maximum value) of their rescaled consistency indices, and the third with implied weights [34] (figure 1; electronic supplementary material, figure S1). The single tree yielded by the implied weights run (figure 1; electronic supplementary material, figure S3) was chosen for all subsequent analyses for three reasons. First, it is better resolved than the strict consensus of the shortest trees from the initial parsimony analysis that used unweighted and unordered characters (see electronic supplementary material, figure S1*a*). Second, it fits well with the agreement subtree yielded by those trees (electronic supplementary material, figure S1*b*). Third, it supports patterns of relationships that are consistent with those from the most recent studies [29,33]. The selected tree was time-calibrated with the methods presented in [35] and their subsequent implementations in references [36,37] in order to obtain branch durations in million years. The time-calibration codes are available at http://www.graemetlloyd.com/methdpf.html, and operate in the R statistical environment (http://www.r-project.org). The time-calibrated tree (see electronic supplementary material, figure S3*a*) is reported in Newick format in the electronic supplementary material. The list of state changes along the tree branches was obtained under the accelerated (ACCTRAN) and delayed (DELTRAN) transformations (respectively, placing state changes as close to, and as far away from, the tree root as possible). All analyses were executed in TNT [38] (for parsimony runs with equally weighted characters and implied weights) and PAUP* [39] (for parsimony runs with reweighted characters and for the output of ACCTRAN and DELTRAN changes).

### (b) Multivariate treatment of inter-taxon distances

We obtained pairwise generalized Euclidean distances (electronic supplementary material, dataset S4) from the cladistic data in the software Matrix [36,40–42], subjecting them to principal coordinates (PCo) analysis in the R ape package [43]. We used the scores (i.e. coordinates) of the taxa along the first 20 PCo axes (see electronic supplementary material, dataset S5) for all disparity analyses (see electronic supplementary material, datasets S6 and S7).

### (c) Morphospace analyses

**Figure 2.**
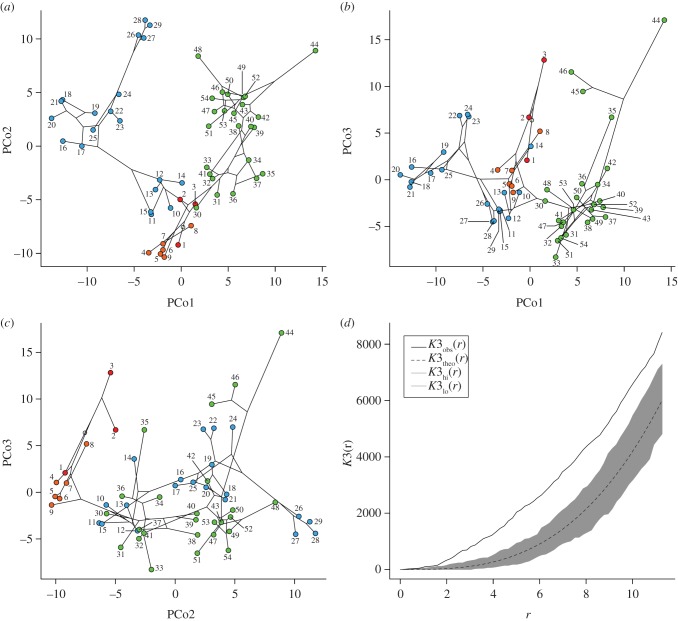
Cynodont morphospace, with taxon identification numbers as in figure 1. (*a*–*c*) Phylomorphospace two-dimensional plots using the PCo1–3 axes. (*d*) *K* function (solid line) for taxon distribution in the three-dimensional space delimited by axes PCo1–3; the dashed line is the theoretical *K* function for a random 54-point distribution; the grey area is the confidence envelope; the *r* distances on the horizontal axis are dimensionless. (Online version in colour.)

We built phylomorphospace [44–47] (i.e. phylogenies superimposed on morphospace) in the planes delimited by combinations of the first three PCo axes (figure 2*a–c*), with internal node positions estimated via maximum likelihood using the R phytools package [47]. To examine patterns of taxon distribution in morphospace, we applied Ripley's *K* function [48] (using the R spatstat package [49]) to the taxon distribution in the three-dimensional space delimited by PCo axes 1–3. Ripley's *K* function quantifies spatial homogeneity by calculating the mean probability of encountering neighbouring data points further away from any given point and for increasing distances from the latter (see also additional explanation in the electronic supplementary material). The *K* function associated with the taxon distribution in morphospace was plotted alongside the *K* function associated with a random (Poisson) process of data point addition in an appropriately sized three-dimensional ‘observation box’ [50]. The theoretical *K* function has the same number of points as our taxon set. If the *K* function associated with the observed distribution of taxa occurs above (respectively, below) the theoretical *K* function along some distance scales, then the taxa are more (respectively, less) clustered on those distances than expected from the Poisson process. Significant differences between observed and theoretical distributions were assessed through Monte Carlo simulations of 999 random 54-point distributions. These distributions were used to build a confidence ‘envelope’ around the theoretical *K* function [49] (figure 2*d*). We point out that the distances along the horizontal axis of the *K* function plot are dimensionless. The exploration of null models of taxon distribution in morphospace is beyond the scope of this paper, and we propose to investigate it in depth as a separate exercise in which we test spatial heterogeneities in the light of Brownian models of taxon branching.

To assess the extent to which relative positions of taxa in morphospace mirror patterns of taxon distribution on the tree, we quantified the degree and significance of the correlations between pairwise generalized Euclidean distances and square-root-transformed phylogenetic distances derived from branch durations [51]. Correlations were assessed through Mantel tests [52] using Spearman's *ρ*, Kendall's *τ* and Pearson's *r*. The probabilities associated with each of these coefficients were obtained via 999 random permutations of the matrix structure. The Mantel test was not used to detect phylogenetic signal. It was used solely to evaluate the degree of linear dependence (i.e. the strength and significance of the correlation) between two distance sets. A Mantel test is eminently suitable in this context, because the branch durations were obtained with information on taxon ages only [37], independent of character changes [35], although note that the branching pattern itself is based on the character data. We recall that the correlation coefficient measures the ‘ability’ of the two distance sets to change in a corresponding manner, whereas the *p*-value is the probability of obtaining that coefficient when the two sets are uncorrelated. A significant *p*-value may be found even with a low correlation. In the electronic supplementary material, we examine phylogenetic signal briefly.

### (d) Disparity analyses

We calculated four disparity indices—namely sum and root-product of ranges (measuring amount of morphospace occupation), and sum and root-product of variances (measuring the dispersal of taxa relative to the centroid of their own group)—in the software Rare [40–42]. For each index and taxon set (with taxa grouped according to major groups and intervals), we built unrarefied and rarefied ‘profiles’ of mean disparity values and associated 95% CIs (from 1000 bootstrap replicates). Rarefied values were based on the smallest of any groups (either systematic or temporal).

**Figure 3.**
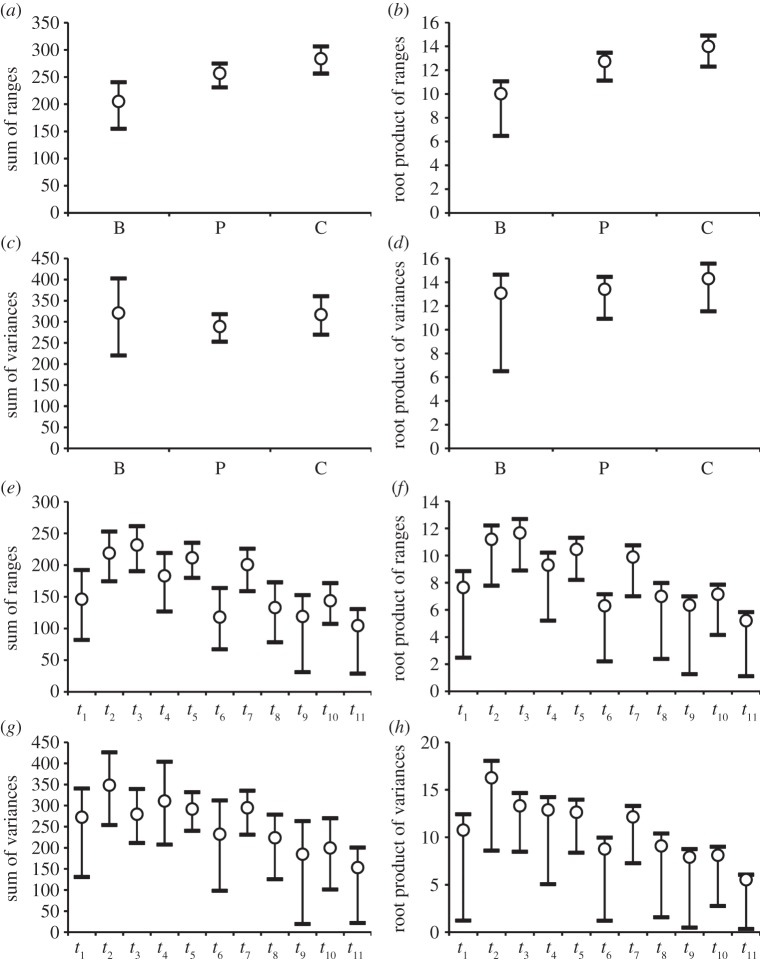
Unrarefied mean disparity with 95% CIs for four indices. (*a*–*d*) Group disparity (B, basal taxa; C, cynognathians; P, probainognathians). (*e*–*h*) Temporal disparity in intervals *t*_1_–*t*_11_ (see main text).

For disparity calculations (figure 3; electronic supplementary material, figure S2), we included solely terminal taxa [40–43], and no internal nodes. A discussion of recently proposed methods [53] that include internal nodes will be dealt with in a separate work. Mean disparity values for each index were calculated for three groups, that is: a paraphyletic array of basal taxa (*Charassognathus* to *Platycraniellus*); monophyletic Cynognathia (*Cynognathus* to *Exaeretodon*); and monophyletic Probainognathia, inclusive of mammals (*Lumkuia* to *Bienotherium*; figure 1). Statistical differences among the distributions of the three groups in morphospace were assessed by subjecting the PCo scores on the first 20 axes to non-parametric multivariate analysis of variance (npMANOVA; testing for similarities in the distribution of the groups’ variances) [54] and non-parametric analysis of similarity (ANOSIM; testing for equal median and range values of ranked dissimilarities in the groups) [55]. Both analyses were run in PAST [56]. For disparity through time, we used stratigraphic stages or substages [13,57] (durations in Myr and abbreviations in brackets): *t*_1_, Wuchiapingian–Changhsingian (5; WUC–CH); *t*_2_, Induan–Olenekian (7; IND–OLE); *t*_3_, Anisian (4; ANS); *t*_4_, Ladinian (6; LAD); *t*_5_, Carnian (7; CRN); *t*_6_, Early Norian (4; NOR); *t*_7_, Middle Norian (12); *t*_8_, Late Norian–Rhaetian (10; RHT); *t*_9_, Hettangian (5; HET); *t*_10_, Sinemurian (7; SIN); *t*_11_, Pliensbachian (7; PLB). For each time interval, the following pairs of numbers refer to taxa in the tree followed by total recorded taxa: *t*_1_, (5/7); *t*_2_, (9/9); *t*_3_, (14/19); *t*_4_, (7/8); _t5,_ (11/12); *t*_6_, (4/8); *t*_7_, (9/12); *t*_8_, (5/10); *t*_9_, (5/5); *t*_10_, (7/12); *t*_11_, (5/8). Intervals with low or no known diversity were combined with adjacent intervals.

### (e) Rate analyses

For analyses of evolutionary rates [35], trees with branch lengths representing the number of character-state changes under ACCTRAN and DELTRAN optimizations were built. These are presented in Newick format in the electronic supplementary material. These numbers were corrected for missing data, following recommendations in references [35–37], by considering the number of characters for which species could actually be scored (patristic dissimilarity [37,41,58]). The rates are calculated as the ratio between the corrected number of changes on the branches and the branch durations. Newick formats of the tree, with branch lengths expressed as rates, are given in the electronic supplementary material, figure S3*b*,*c* (for the distribution of rates by groups and through time, see electronic supplementary material, dataset S8).

**Figure 4.**
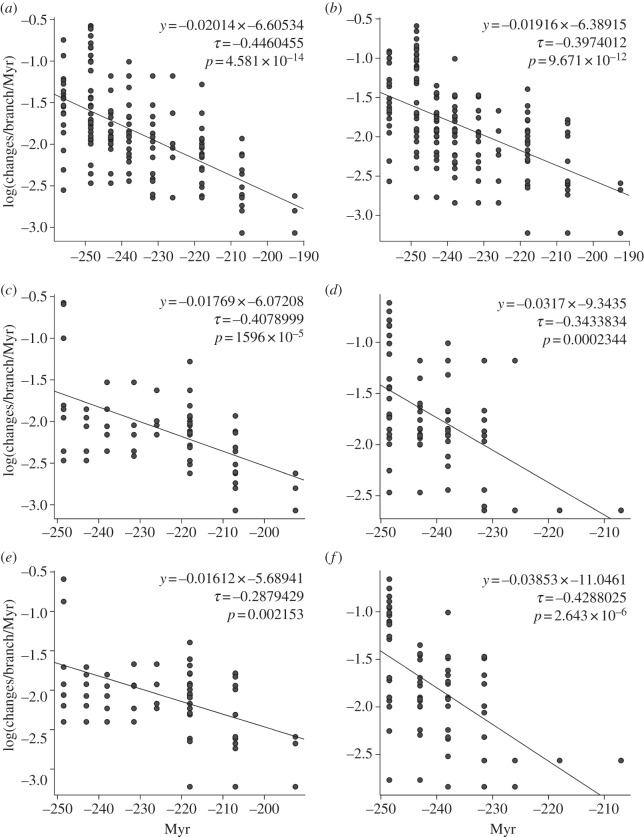
Plots of rates versus time, including regression line and equation, and the strength and significance of Kendall's correlation. (*a*) ACCTRAN and (*b*) DELTRAN rates for all taxa. (*c*,*d*) ACCTRAN rates for (*c*) probainognathians and (*d*) cynognathians. (*e*,*f*) DELTRAN rates for (*e*) probainognathians and (*f*) cynognathians.

Pragmatically, we assume that the changes on the terminal branches (phylogeny-derived autapomorphies of taxa) had already accrued before the earliest occurrences of taxa, such that no further changes can logically have taken place during the observed range of any taxon (the observed range is the time period between the earliest and last documented occurrences). We further assume for the sake of simplicity that rates do not change throughout the duration of a branch. We reserve a discussion of rate heterogeneities for a separate paper (however, see [37]). The rates were grouped based upon tree shape (one set per major cynodont group) and time (one set per interval). Intervals correspond to those for disparity analyses up to the Rhaetian; we grouped Jurassic stages together given the paucity of branches with realized rates (figures 1 and 4).

We evaluated the significance of rate differences in the three groups through a Kruskal–Wallis test and *post hoc* Mann–Whitney pairwise tests in PAST [56]. Using the midpoints of the nine intervals, we examined rate trends through time. If a branch crossed several intervals, we assigned the rate value associated with that branch to all relevant intervals. We quantified the relationship between rates and intervals via linear regression in R, evaluating its strength and significance with Kendall's rank-order correlation [35]. We further examined changes in rate through time in each of the two eucynodont clades (the basal taxa were not considered as they occur in a single interval). Finally, we used recent protocols for testing the equality of all branch rates with likelihood ratio tests, and for detecting significantly high or low rate values [37] under both ACCTRAN and DELTRAN. The few zero-rate branches were excluded from all analyses, and non-zero rates were log_10_-transformed prior to calculations [35].

### (f) Correlations between diversity and disparity

For each of the four disparity indices, we compared the unrarefied mean values in the *t*_1_–*t*_11_ time bins with the number of cynodonts actually used in the disparity calculations, as well as with the total number of described cynodonts (see electronic supplementary material, figure S4). It may be argued that the latter comparison is unwarranted, as not all taxa have been considered in the disparity analyses. However, many excluded taxa are very fragmentary and mostly consist of incomplete jaws or teeth. Their position in morphospace is likely to reflect proximity to taxa with which they share the greatest similarities in coded characters [40]. However, we reserve a proper test of this assumption in the light of a comprehensive taxon set (work in progress).

## 3. Results

### (a) Phylogeny

Cynodont relationships match closely those retrieved in some recent analyses (see electronic supplementary material) [29,33]. When plotted against geological time, the tree shows four lineages crossing the PTB: two for Galesauridae (lineages leading to *Galesaurus* and *Progalesaurus*), one for *Thrinaxodon*, and one subtending *Platycraniellus* and eucynodonts. The separation between Cynognathia and Probainognathia occurred early in the Triassic. Cynognathians disappeared in a stepwise fashion in the Ladinian and Late Triassic, but probainognathians continued to diversify, giving rise to tritylodonts, tritheledonts and mammals (figure 1).

### (b) Correlations among distance matrices

Mantel tests show that morphological distances correlate weakly with phylogenetic distances (*ρ* = 0.3489; *τ* = 0.2329; *r* = 0.3251); all correlation coefficients differ significantly from zero (*p* = 0.001). To assess whether these results might be generated by branches with unusually long durations (e.g. in probainognathians), we ran additional Mantel tests following deletion of the morphological and phylogenetic distances that pertain to each of the major groups in turn. When basal taxa were excluded, correlations improved slightly, but were still moderate to weak (*ρ* = 0.4425; *τ* = 0.2934; *r* = 0.4038; in all cases, *p* = 0.001). With the exclusion of cynognathians, all correlations became marginally stronger than in the original calculations with all distances (*ρ* = 0.5451; *τ* = 0.3813; *r* = 0.4963; in all cases, *p* = 0.001). The exclusion of probainognathians had the most profound effects, with correlations becoming very weak and also non-significant in one case (*ρ* = 0.2162, *p* = 0.016; *τ* = 0.1467, *p* = 0.012; *r* = 0.1395, *p* = 0.07). These results can be explained by noting that cynognathians reveal many instances of discordant patterns (more numerous than in basal taxa and probainognathians) between the branching order of taxa and their positions in morphospace, as shown by intersecting branches (terminal and internal) in the phylomorphospace plots (figure 2*a–c*).

### (c) Patterns of morphospace occupation

The distribution of all cynodonts in the three-dimensional space delimited by the first three PCo axes both departs from random and shows significant clustering on all distance scales (figure 2*d*). This clustering reflects phylogenetic proximity in some, but not all cases. Certain clusters include taxa from widely separated parts of the tree (either within each of the groups or from different groups). As an example, mammals (circles 24 and 25) are sister group to tritylodonts (circles 26–29), but close to tritheledonts (circles 17–21) in all plots (figure 2*a–c*). As an additional example, *Probainognathus* (circle 14) is close to *Dvinia* (circle 2). The three major groups are significantly separate in morphospace (npMANOVA: *F* = 4.672; *p* = 0.0001; ANOSIM: *R* = 0.4153; *p* = 0.0001; for all pairwise *post hoc* tests, *p* = 0.0003).

The cynognathian radiation corresponds to the acquisition of several apomorphies; for example, a deep zygomatic arch, a suborbital jugal process for the masseter jaw-closing muscle, and a deep squamosal groove to aid sound conduction to the middle ear [14,16]. This radiation was marked by a less heterogeneous distribution of taxa in morphospace than probainognathians, but a greater prevalence of discordant patterns with phylogeny. Probainognathians showed a more heterogeneous distribution in morphospace occupation, presumably underpinned by specializations (e.g. dietary) of three clades—tritheledonts, tritylodonts and mammals—but fewer discordant patterns. At present, it is not possible to ascertain whether these patterns reflect real differences between these two clades or a prevalence of cynognathians in the data matrix. One possibility is that cynognathians may be characterized by overall higher levels of homoplasy than probainognathians, and this is borne out by the wide range of specializations in the former compared with the latter. However, a more stringent test of this hypothesis must await the discovery of additional taxa and the construction of a new and expanded dataset.

### (d) Disparity

The mean disparity values for the three major groups are shown in figure 3*a*–*d* (unrarefied values) and electronic supplementary material, S2*a*–*d* (rarefied values). If we consider the mean values first (disregarding for a moment the confidence intervals), then we see similar disparity ‘profiles’ for the unrarefied and rarefied plots. With the range-based indices and the root-product of variances, the basal taxa are less disparate than probainognathians, and these are less disparate than the cynognathians. Differences in mean disparity between any two groups are smaller in the case of the rarefied range indices (the root-product of variances is affected only slightly by rarefaction) than they are for the corresponding unrarefied indices. With both the rarefied and the unrarefied sum of variances, basal taxa and cynognathians exhibit comparable mean values that are slightly higher than the value for probainognathians. A significant difference in mean values—based on non-overlap between confidence intervals [59]—occurs between cynognathians and basal taxa for the two unrarefied range indices, and also between probainognathians and basal taxa for the root-product of ranges only. However, in all other cases (including for the unrarefied variance indices), confidence intervals show various degrees of overlap. Rarefaction produces non-significant pairwise differences between groups for all indices.

The unrarefied and rarefied disparity plots through time are illustrated in figures 3*e*–*h* and electronic supplementary material, S2*e–h*. Unrarefied profiles of mean disparity show similar trends from Early Norian (*t*_6_) to Pliensbachian (*t*_11_) for all four indices, with a peak during the Middle Norian (*t*_7_). From *t*_1_ (Wuchiapingian + Changhsingian) to *t*_5_ (Carnian), however, the profiles of the range indices differ from those of the variance indices. With both range indices, mean disparity increases from Wuchiapingian + Changhsingian to Anisian (*t*_3_), drops in the Ladinian (*t*_4_), increases again in the Carnian (*t*_5_), and reaches its minimum at the Carnian–Early Norian transition. With the variance indices, mean disparity drops across the Early–Middle Triassic transition (*t*_2_–*t*_3_), but changes very little throughout the Middle and early Late Triassic (*t*_3_–*t*_5_). In the Induan–Olenekian (*t*_2_), Anisian (*t*_3_) and Carnian (*t*_5_), the mean values for the sum of ranges are significantly separate from the values in each of the following intervals: Early Norian (*t*_6_), Late Norian–Rhaetian (*t*_8_), Hettangian (*t*_9_), Sinemurian (*t*_10_) and Pliensbachian (*t*_11_). In addition, a significant difference characterizes Middle Norian (*t*_7_) versus Hettangian and Middle Norian versus Pliensbachian. With the unrarefied sum of variances, the only significant differences are for Pliensbachian versus each of the following time intervals: Induan–Olenekian, Anisian, Ladinian, Carnian and Middle Norian. The patterns exhibited by the two root-products are very similar to those of the two sums. With rarefaction, all pairwise comparisons between time intervals become non-significant, although the profiles of mean disparity change little. These results may imply potential sampling biases in the cynodont record, but we note that the subsampling routines are contingent upon the Early Norian four-taxon sample. With this small sample, it is not surprising that the rarefied plots show hardly any instance of significant differences.

Following the Triassic–Jurassic extinction, cynodont disparity stabilized around levels comparable with the Early Norian (or slightly lower). Such values also compare well with mean Permian values for all indices. With the two range indices and the root-product of variances, mean disparity attains its highest values from Induan–Olenekian to Carnian as well as in the Middle Norian, before the origin of mammals.

### (e) Rates

Both ACCTRAN- (figure 4*a*) and DELTRAN-based (figure 4*b*) rates decreased through time, with a moderate negative correlation between rates and time. Probainognathians (figure 4*c,e*; ACCTRAN/DELTRAN) and cynognathians (figure 4*d,f*; ACCTRAN/DELTRAN) exhibit a moderate (figure 4*c,f*) to very weak (figure 4*d,e*) negative correlation. Rate decrease through time is not related to particular portions of cynodont phylogeny and is not altered by different character-state optimizations.

Global tests of differences in the three groups show significant results with ACCTRAN (Kruskal–Wallis test: *H* = 28.58; *p* < 0.001) and DELTRAN (Kruskal–Wallis test: *H* = 18.66; *p* < 0.001) rates. Mann–Whitney tests show that rates differ between basal taxa and probainognathians (ACCTRAN: *p* < 0.001; DELTRAN: *p* < 0.001) as well as between cynognathians and probainognathians (ACCTRAN: *p* < 0.001; DELTRAN: *p* < 0.001), but there is no significant difference between cynognathians and basal taxa.

Likelihood ratio tests reject the null hypothesis of equal branch rates across the tree for both ACCTRAN and DELTRAN rates 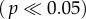. Under both optimizations, significant rate heterogeneities were detected. Green and red circles indicate significantly high and low rates, respectively, in electronic supplementary material, figure S3*b*,*c* (branch lengths equivalent to ACCTRAN and DELTRAN rates). A number of significant rates are common to both the ACCTRAN and the DELTRAN rate trees. High rates occur on the internal branches of basal epicynodonts and several (except the most basal) internal branches of both eucynodont clades, but no significant rates are detected immediately before the cynognathian–probainognathian split. Significantly high rates occur in many of the proximal internal branches of cynognathians. However, there are no significant rates in most internal branches of probainognathians, including close to the ancestral node of mammals; but note the significantly high rate value along the branch leading to the mammals’ sister group—the tritylodonts. Such a high rate is presumably due to the array of anatomical specializations of tritylodonts, including their superficially rodent-like dentition with elongate incisors and long molars with multiple rows of cusps, and their mammal-like pelvis and femur. Low rates are concentrated mostly on the terminal branches of both eucynodont clades.

### (f) Diversity and disparity through time

When only the taxa included in the phylogeny are considered, there is a significant correlation between diversity and un-rarefied disparity during the *t*_1_–*t*_11_ time intervals for all indices and without generalized differencing of time series [37], and for the range indices with generalized differencing (see electronic supplementary material, dataset S12). When total cynodont diversity through time is considered, then a significant correlation characterizes only range indices, regardless of generalized differencing. Diversity rises and drops track disparity closely with range indices. Discordant diversity and disparity curves in *t*_1_–*t*_5_ occur with the variance indices (see electronic supplementary material, figure S4). Again, these results may reflect, at least in part, unequal sample sizes in different time intervals, as well as changes in the quality of the cynodont record through time (work in progress).

## 4. Discussion

The tempo and mode of clade diversification are key topics in current macroevolutionary and biodiversity studies. Patterns of diversification following mass extinctions are of great interest, as clades might be expected to expand in a relatively unfettered manner as a result of very low competition. The origin of mammals is a benchmark for testing models of diversification (e.g. long fuse versus early burst [60,61]). As the closest fossil relatives of mammals, cynodonts offer a unique, deep-time perspective on the speciation of stem groups. Using the results from our investigation, we speculate on an ecological scenario that might explain the different models of speciation among eucynodonts.

During the Late Early and Early–Middle Triassic, cynodont diversity was dominated by cynognathians. It was not until the Early Norian that probainognathians became more widely represented. Differences in the relative contributions of the two eucynodont clades to overall cynodont diversity may reflect sampling bias against probainognathians. Probainognathians were mostly small to mid-sized, and their size may have limited their fossilization potential. The long branches of probainognathians appear to be consistent with a preservation bias, but an alternative interpretation for such long branches rests on the observation that several early probainognathians have long stratigraphic ranges (unlike cynognathians), and their overall morphology and dietary adaptations allow one to characterize them as a low-diversity guild of carnivorous/insectivorous generalists. Recent studies of the impact of large-scale crises on the structure and stability of ancient food webs [3,62] have shown that in the earliest Triassic, the faunal community in the *Lystrosaurus* Assemblage Zone (LAZ) of the Karoo Basin were dominated by few herbivores, and numerous, small and intensely competing carnivores and insectivores. It is hypothesized that this intense competition destabilized the community structure of the LAZ. Roopnarine & Angielczyk [62] hypothesized that this lack of stability may have been a key factor in influencing the modalities of tetrapod diversification that eventually led to the Middle Triassic *Cynognathus* Assemblage Zone, where tetrapods became ecologically differentiated into herbivores and omnivores [62].

Cynodont diversification is consistent with this scenario. Thus, cynognathians expanded rapidly in the Early/Middle Triassic, experienced high evolutionary rates between their major branching events, invaded novel ecospace following acquisition of a herbivorous/omnivorous diet and showed a variety of body sizes. Conversely, probainognathians remained at very low diversity until the middle Late Triassic, experienced significantly high rates only in the most basal internal branches, were generally small, and shifted from a carnivorous to a herbivorous and omnivorous diet only in later stages of their history. Community structure may have been instrumental in imposing different evolutionary rates on the two eucynodont clades, whereby cynognathian evolution proceeded at significantly higher rates than in the rest of the tree, whereas probainognathians evolved more slowly.

Despite a steady increase in new discoveries, Mesozoic mammal richness and disparity are relatively low [18,19,28]. This may be consistent with a long fuse model for the radiation of this clade. Some evidence in support of the long fuse model comes from the observation that the two early mammals, *Morganucodon* and *Sinoconodon*, fall well within the region of morphospace occupied by probainognathians. This finding further supports the hypothesis that probainognathians were a slowly evolving and fairly conservative group. However, the radiation of Triassic cynodonts points towards a more elaborate pattern of diversification that may have included an early burst component, thus effectively indicating a complex set of inter-nested diversification models. It is too early to speculate on the accuracy of this pattern, and we are in the process of testing it in the light of a cynodont–Mesozoic mammal dataset. Current debates over the prevalence of one or few models may be partly incomplete. The inclusion of both crown and stem groups in the study of radiations may provide a firm ground for evaluating the impact of fossil diversity on models of clade diversification.
